# Clinical Significance of Lifetime Maximum Body Mass Index in Predicting the Development of T2DM: A Prospective Study in Beijing

**DOI:** 10.3389/fendo.2022.839195

**Published:** 2022-06-02

**Authors:** Xiaomeng Jia, Anping Wang, Longyan Yang, Yu Cheng, Yajing Wang, Jianming Ba, Jingtao Dou, Yiming Mu, Dong Zhao, Zhaohui Lyu

**Affiliations:** ^1^ Department of Endocrinology, The First Medical Center of Chinese PLA General Hospital, Beijing, China; ^2^ Center for Endocrine Metabolism and Immune Disease, Beijing Luhe Hospital, Capital Medical University, Beijing, China

**Keywords:** maximum body mass index, obesity, mellitus, type 2 diabetes mellitus, predictive model, Beijing

## Abstract

**Background:**

Overweight and obesity are well-known risk factors for type 2 diabetes mellitus (T2DM). The effect of the maximum body mass index (BMImax), which indicates the highest body weight before the diagnosis of T2DM, is not fully understood. This study aimed to explore the predictive value of BMImax in the progression of diabetes.

**Methods:**

This prospective study recruited 2018 subjects with normal glucose tolerance in Beijing, China. The subjects were followed up for eight years, and the association between BMImax and glucose outcomes was evaluated.

**Results:**

Ninety-seven of the 2,018 participants developed diabetes by the end of the study. Compared to individuals with normal glucose tolerance, those who developed diabetes were characterized by higher levels of fasting plasma glucose (FPG), 2 h postload glucose (PBG), glycosylated hemoglobin (HbA_1c_), systolic blood pressure (SBP), and low-density lipoprotein cholesterol (LDL-c), a higher prevalence of a familial history of diabetes and a lower level of high‐density lipoprotein cholesterol (HDL-c). Multivariate regression analysis of sex-stratified groups suggested that FPG, HbA_1c_, SBP and familial history of diabetes were independent risk factors for diabetes, but that BMImax was a unique indicator for female patients.

**Conclusions:**

BMImax might be an independent predictor of T2DM in females, but it does not seem to be associated with the risk of diabetes in males. BMImax could be regarded as an indicator in the prevention and management of diabetes.

## Introduction

The prevalence of diabetes mellitus in the Chinese population is rapidly increasing, causing China to be the country with the largest population of individuals with diabetes in the world. Type 2 diabetes mellitus (T2DM) is the most common type of diabetes, and its prevalence was reported to be 0.67% in 1980, 11.6% in 2020 and 10.9% according to latest research (1). Approximately 46% of adults in China are obese or overweight, and the prevalence of T2DM in overweight and obese populations is 12.8% and 18.5%, respectively (2). Obesity and T2DM have common triggers in China, such as rapid urbanization and lifestyle changes (3, 4). Obesity can lead to impaired glucose tolerance and insulin resistance (5, 6). Recently, it has been reported that the influence of body weight on T2DM is more obvious among Asian people (7). A nationwide prospective study suggested that insulin resistance was significantly correlated with T2DM, more so than with β-cell dysfunction, in Chinese adults, and that this association pattern was more prominent among those with obesity (8). Body mass index (BMI) is the preferred factor for evaluating adiposity. A strong positive association between BMI and an elevated risk of T2DM has been widely reported by previous studies (9–12). However, the research mostly focused on current BMI or BMI at baseline. Life-time maximum body mass index (BMImax) is an indicator of BMI history and has been reported to be a screening tool for predicting the function of β-cells at the time of diagnosis and reflecting the period when obesity more obviously affected blood glucose (13). Although the associations between BMImax and hypertension (14), fatty liver (15), chronic kidney disease (16) and cardiovascular disease (17) has been reported, less is known about the effect of BMImax on the onset of T2DM.

We hypothesized that BMImax, as an index independent of any intervention, such as medication and lifestyle management, might have a marked association with T2DM on the progression of diabetes. To further understand the effect of obesity on T2DM, we conducted an epidemiological study in a Chinese population that was followed up for 8 years.

## Methods

### Study Population

The study, which was designed to assess the related factors of diabetes enrolled 6,497 participants who were over 40 years old in the community of Shou Gang, Beijing, in 2011, and conducted comprehensive interviews in 2018 after 8 years from baseline. At baseline, 75-g oral glucose tolerance test (OGTT) results for 3885 of the 6497 participants showed normal glucose tolerance. Of the 3885 subjects, 8 patients who had been diagnosed with diabetes before the BMImax period, 716 patients with missing visits, 623 patients who could not accurately recall their historical weight and 520 patients with missing values for blood glucose or other clinical information were excluded, and 2,018 participants were ultimately recruited. The research was approved by the Clinical Research Ethics Committee of the People’s Liberation Army General Hospital, Beijing, China. All participants were fully informed of the study protocol and signed informed consent forms.

### Data Collection

The data were obtained by standardized questionnaire surveys, anthropometric measurements and laboratory examinations, and all the investigators were formally trained. Investigators conducted face-to-face interviews with participants using detailed questionnaires to obtain information on sociodemographic characteristics, lifestyle, medical history, and current dietary regimen. Participants who smoked at least 1 cigarette a day or 7 per week over 6 months were defined as regular smokers. Alcohol drinking was claasified as current drinking (at least once per week for six months or longer), former drinking now abstaining and never drinking.

All subjects underwent standard anthropometric measurements, including body height (BH), body weight (BW), waist circumference (WC), hip circumference (HC), and blood pressure, by the same trained investigators. The subjects were required to remove their shoes and to wear light clothes. BH and BW were recorded to the nearest 0.1 cm and 0.1 kg, respectively. BMI was calculated by the formula: BW/BH^2^ (kg/m^2^). The BMImax was calculated based on the highest weight the participants could recall. Blood pressure was measured 3 times at 5-minute intervals after the participants had rested in a seated position for 15 minutes. Average values of systolic blood pressure (SBP), diastolic blood pressure (DBP) and heart rate (HR) were obtained.

Blood samples were collected in the morning, after the participants had fasted for more than 8 h overnight. A 75-g OGTT was performed for participants who had not been previously diagnosed with diabetes. Blood samples were sent to central laboratories for testing. Fasting plasma glucose (FPG) and 2 h postload glucose (PBG) were measured by a hexokinase assay, and glycosylated hemoglobin (HbA_1c_) was assessed by ion-exchange high-pressure liquid phase assay. High-density lipoprotein cholesterol (HDL-c), low-density lipoprotein cholesterol (LDL-c), total cholesterol (TC), serum triglyceride (TG), creatinine (Cr) and other biochemical indicators were measured by a Roche biochemical analyzer (Cobas 8000 Modular Analyzer Series, Roche Diagnostics, Basel, Switzerland).

### Variable Definitions

According to the Guidelines for Prevention and Control of Overweight and Obesity in Chinese Adults, participants were stratified into BMI groups as follows: underweight (BMI<18.5 kg/m^2^), normal weight (18.5≤BMI ≤ 24.0 kg/m^2^), overweight (24.0≤BMI<28.0 kg/m^2^) and obese (BMI≥28 kg/m^2^). Diabetes was diagnosed based on the criteria recommended by the World Health Organization (WHO): FPG≥7.0 mmol/l, 2 h PBG≥11.1 mmol/l, or self-reported history of diabetes diagnosed by physicians or undergoing hypoglycemic therapy with antidiabetic medications.

### Statistical Analysis

All analyses were performed by SAS version 9.2 (SAS Institute, Inc., Cary, NC, USA) and R version 3.02. Categorical variables are described by the frequency and prevalence, and the chi-square test or Fisher’s exact probability method was used for comparisons between groups. Continuous variables with a normal distribution were described as the mean ± standard deviation (SD), and differences between groups were analyzed by ANOVA. Normally distributed continuous variables are described as the median (25^th^ percentile-75th percentile), and differences between groups were analyzed by the Wilcoxon rank-sum test. Unconditional multiple logistic regression analysis and bilateral tests were used to investigate factors related to diabetes. The nomogram function in R software was used to visualize the prediction model, and the receiver operating characteristic (ROC) curve was used to evaluate the efficiency of the prediction model. *P* < 0.05 was considered indicative of statistically significance.

## Results

### Clinical Characteristics of Subgroups Stratified by Glucose Outcomes at Baseline

A total of 2,018 subjects were enrolled in the study, consisting of 1,386 females and 632 males, and the female-to-male ratio was 2.2:1. The mean age of the participants was 54.18 ± 6.98 years. At the end of the 8-year follow-up period, 97 of 2,018 subjects had developed diabetes, and 1,921 still maintained normal glucose tolerance. Based on the results of the 75-g OGTT, all subjects were divided into normal glucose and diabetes groups. The clinical characteristics were comparatively analyzed. Compared with the subjects with normal glucose tolerance, those who developed diabetes had a higher BMImax (27.02 ± 4.03 kg/m^2^ vs 25.66 ± 3.38 kg/m^2^, *P*<0.001), and the prevalence of obesity defined by BMImax was also higher (37.11% vs. 21.13%, *P*=0.002). Those diabetic patients presented with higher baseline BMI (26.05 ± 3.73 kg/m^2^ vs. 25.25 ± 3.26 kg/m^2^, *P*=0.020), waist-to-hip ratio (0.89 ± 0.06 vs. 0.88 ± 0.06, *P*=0.019), fasting plasma glucose (5.52 mmol/l ±0.33 mmol/l vs. 5.28 ± 0.38, *P*<0.001), 2 h postload glucose (6.44 ± 0.99 mmol/l vs. 6.06 ± 1.02 mmol/l, *P*<0.001), HbA_1c_ (6.02 ± 0.42% vs. 5.75 ± 0.40%, *P*<0.001), SBP (134.47 ± 15.42 mmHg vs. 128.36 ± 18.68 mmHg, *P*<0.001), LDL-c (3.40 ± 0.80 mmol/l vs. 3.19 ± 0.81 mmol/l, *P*=0.012), and familial history of diabetes (43.30% vs. 24.26%, *P*<0.001) and lower HDL-c (1.38 ± 0.33 mmol/l vs. 1.50 ± 0.38 mmol/l, *P*=0.003) (**Table 1**).

**Table 1:** Clinical characteristics in subgroups stratified by glucose outcomes at baseline.

Variables	Whole population	Normal	Diabetes	P value
	N = 2018	N = 1921	N = 97	
Age, years	54.18 ± 6.98	54.14 ± 7.02	55.00 ± 6.21	0.238
Sex, N (%)				0.423
Male	632 (31.37)	597 (31.12)	34 (35.05)	
Female	1386 (68.63)	1324 (68.88)	63473 (64.95)	
Waist to hip ratio	0.88 ± 0.06	0.88 ± 0.06	0.89 ± 0.06	0.019
BMI baseline, kg/m^2^	25.29 ± 3.29	25.25 ± 3.26	26.05 ± 3.73	0.020
BMI baseline subgroups, N (%)				0.034
<24 kg/m^2^	716 (35.55)	690 (35.99)	26 (26.80)	
24-28 kg/m^2^	913 (45.13)	867 (45.02)	46 (47.42)	
≥28 kg/m^2^	389 (19.32)	364 (18.99)	25 (25.78)	
BMImax, kg/m^2^ N (%)	25.72 ± 3.43	25.66 ± 3.38	27.02 ± 4.03	0.000
BMImax subgroups, N (%)				0.002
<24 kg/m^2^	617 (30.57)	596 (31.03)	21 (21.65)	
24-28 kg/m^2^	959 (47.52)	919 (47.84)	40 (41.24)	
≥28 kg/m^2^	442 (21.91)	406 (21.13)	36 (37.11)	
Fasting plasm glucose, mmol/l	5.29 ± 0.38	5.28 ± 0.38	5.52 ± 0.33	0.000
2h post load glucose, mmol/l	6.08 ± 1.02	6.06 ± 1.02	6.44 ± 0.99	0.000
HbA_1C_, %	5.76 ± 0.41	5.75 ± 0.40	6.02 ± 0.42	0.000
SBP, mmHg	128.65 ± 18.58	128.36 ± 18.68	134.47 ± 15.42	0.000
DBP, mmHg	74.84 ± 15.57	74.78 ± 15.81	76.08 ± 9.58	0.212
TC, mmol/l	5.24 ± 0.97	5.23 ± 0.97	5.41 ± 0.92	0.075
TG, mmol/l	1.37 ± 0.85	1.37 ± 0.85	1.50 ± 0.75	0.131
HDL-c, mmol/l	1.49 ± 0.38	1.50 ± 0.38	1.38 ± 0.33	0.003
LDL-c, mmol/l	3.20 ± 0.81	3.19 ± 0.81	3.40 ± 0.80	0.012
Family history of diabetes, N (%)	515 (25.52)	473 (24.62)	42 (43.30)	<0.001
Alcohol drinking, N (%)				0.062
Never	1761 (87.26)	1688 (87.87)	84 (86.60)	
Former	43 (2.13)	35 (1.82)	2 (2.10)	
Current	214 (10.61)	198 (10.31)	11 (11.30)	
Smoking status, N (%)				0.094
Never	1668 (82.99)	1593 (83.27)	75 (77.32)	
Former	49 (2.44)	48 (2.51)	1 (1.03)	
Current	293 (14.58)	272 (14.22)	21 (21.65)	

Continuous variables are expressed as mean ± SD and categorical variable are reported for frequency (%). BMI, Body mass index; BMImax, Maximum body mass index; HbA_1C_, Glycosylated hemoglobin; SBP, Systolic blood pressure; DBP, Diastolic blood pressure; TC, Cholesterol; TG, Triglyceride; HDL-c, High‐density lipoprotein cholesterol; LDL-c, Low-density lipoprotein cholesterol.

### Factors Associated With the Development of Diabetes Stratified By Sex

The results of the logistic regression analysis suggested that obesity defined by BMImax was associated with a higher risk of diabetes [2.52 (1.45-4.37), *P*=0.001]. In addition, fasting plasma glucose, 2 h postload glucose, HbA_1c_, SBP, LDL-c, and familial history of diabetes were also associated with an increased risk of diabetes. HDL-c was negatively associated with diabetes. In the multivariate regression model, we adjusted for confounding factors including age, sex, waist-to-hip ratio, baseline BMI, BMImax, SBP, fasting plasma glucose, 2 h postload glucose, HbA_1c_, HDL-c, LDL-c, and familial history of diabetes. The results showed that obesity defined by BMImax was still a risk factor for diabetes [3.26 (1.42-7.52), *P*=0.001]. In addition, fasting plasma glucose [3.69 (1.93-7.07), *P*<0.001], 2 h postload glucose [1.37 (1.08-1.75), *P*=0.010], HbA_1c_ [2.66 (1.67-4.23), *P*<0.001], SBP [1.03 (1.01-1.05), *P*=0.002], HDL-c [0.48 (0.24-0.97), *P*=0.042] and familial history of diabetes [1.94 (1.25-3.01), *P*=0.003] were all factors related to diabetes.

For the sex-specific analysis, among males, the risk of diabetes increased with the level of fasting plasma glucose [9.89 (3.32-29.5), *P*<0.001], 2 h postload glucose [1.63 (1.14-2.33), *P*<0.001], HbA_1c_ [6.08 (2.81-13.14), *P*<0.001] and familial history of diabetes [2.93 (1.45-5.92), *P*=0.003]. The trends were the same in the adjusted regression model. We did not find an association between BMImax and diabetes among male patients.

Among females, diabetes was positively related to obesity defined by BMImax [2.78 (1.45-5.45), *P*=0.002], fasting plasma glucose [5.08 (2.48-10.4), *P*<0.001], HbA_1c_ [2.78 (1.67-4.65), *P*<0.001], SBP [1.01 (1.00-1.02), *P*=0.022] and familial history of diabetes [2.12 (1.27-3.54), *P*=0.004] and negatively related to HDL-c [0.28 (0.13-0.62), *P*=0.002]. In the adjusted model, obesity defined by BMImax increased the risk of diabetes 3.34 times compared with normal weight [3.97 (1.38-11.44), P=0.011]. Plasma glucose and familial history were common risk factors for diabetes in both males and females. The effect of BMImax on progression to diabetes was more pronounced in females.

### Nomogram Construction and Validation

**Figure 1:**
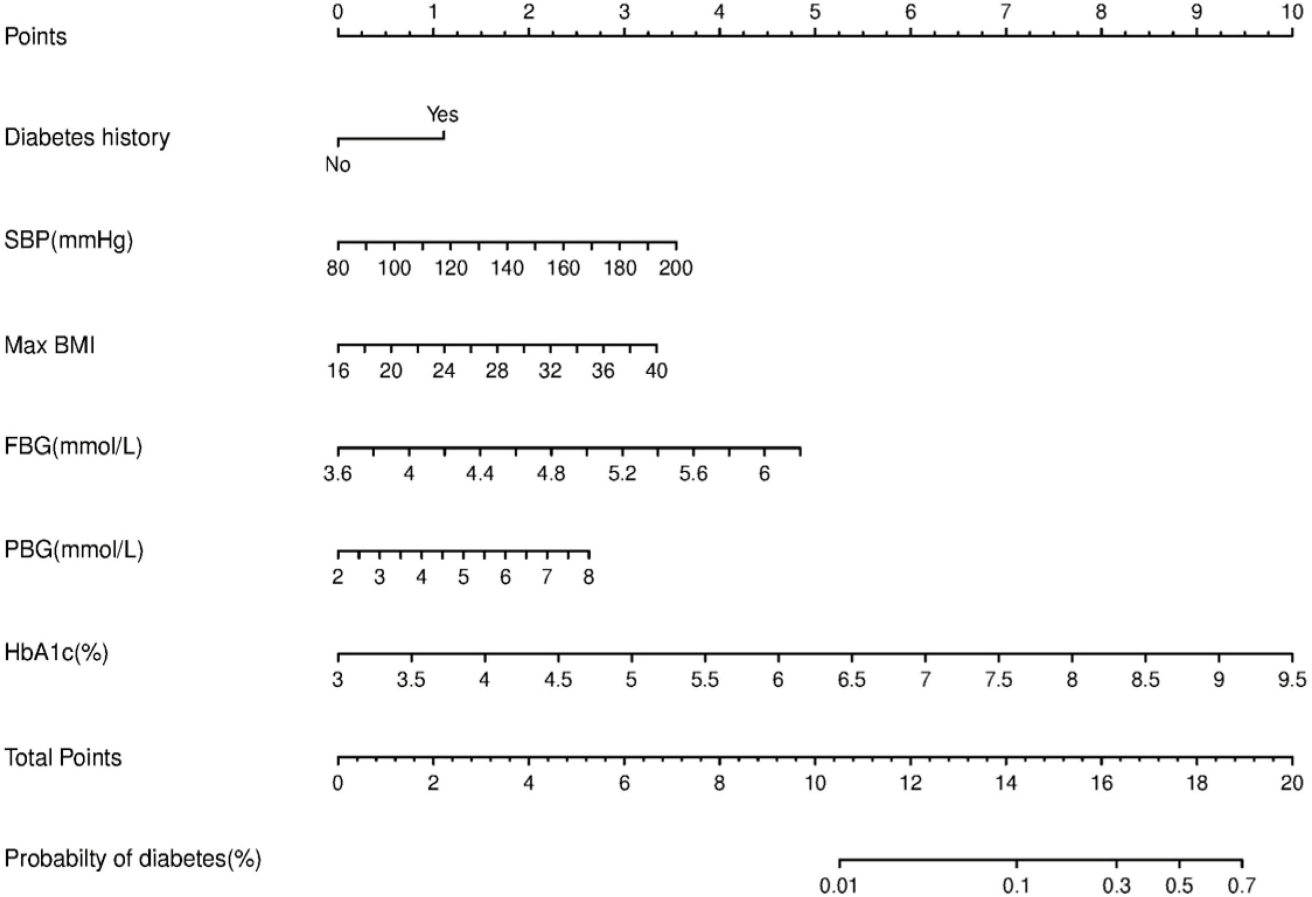
Nomogram predictive model for diabetes.

**Figure 2:**
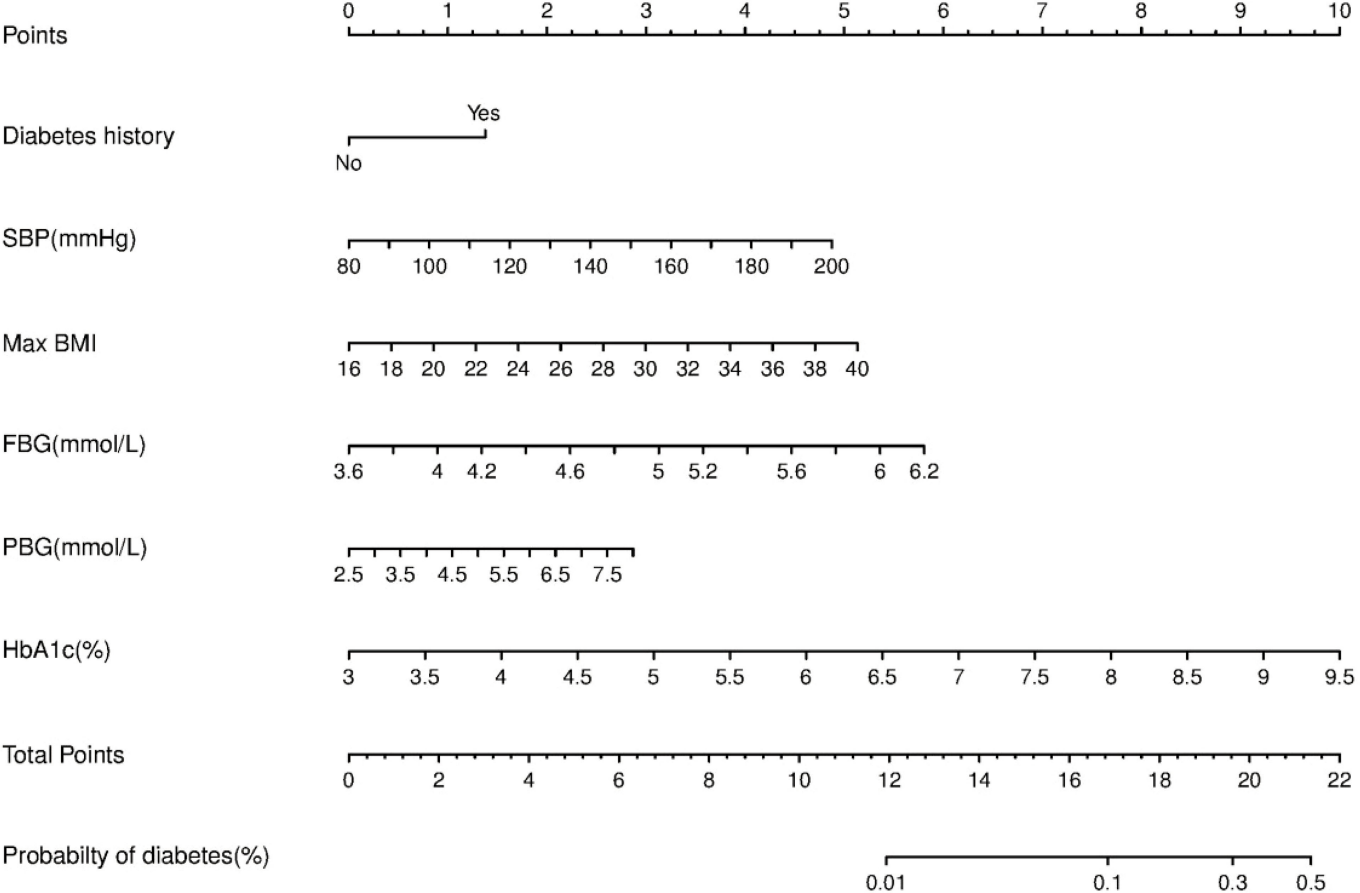
Nomogram predictive model for diabetes in females.

**Figure 3:**
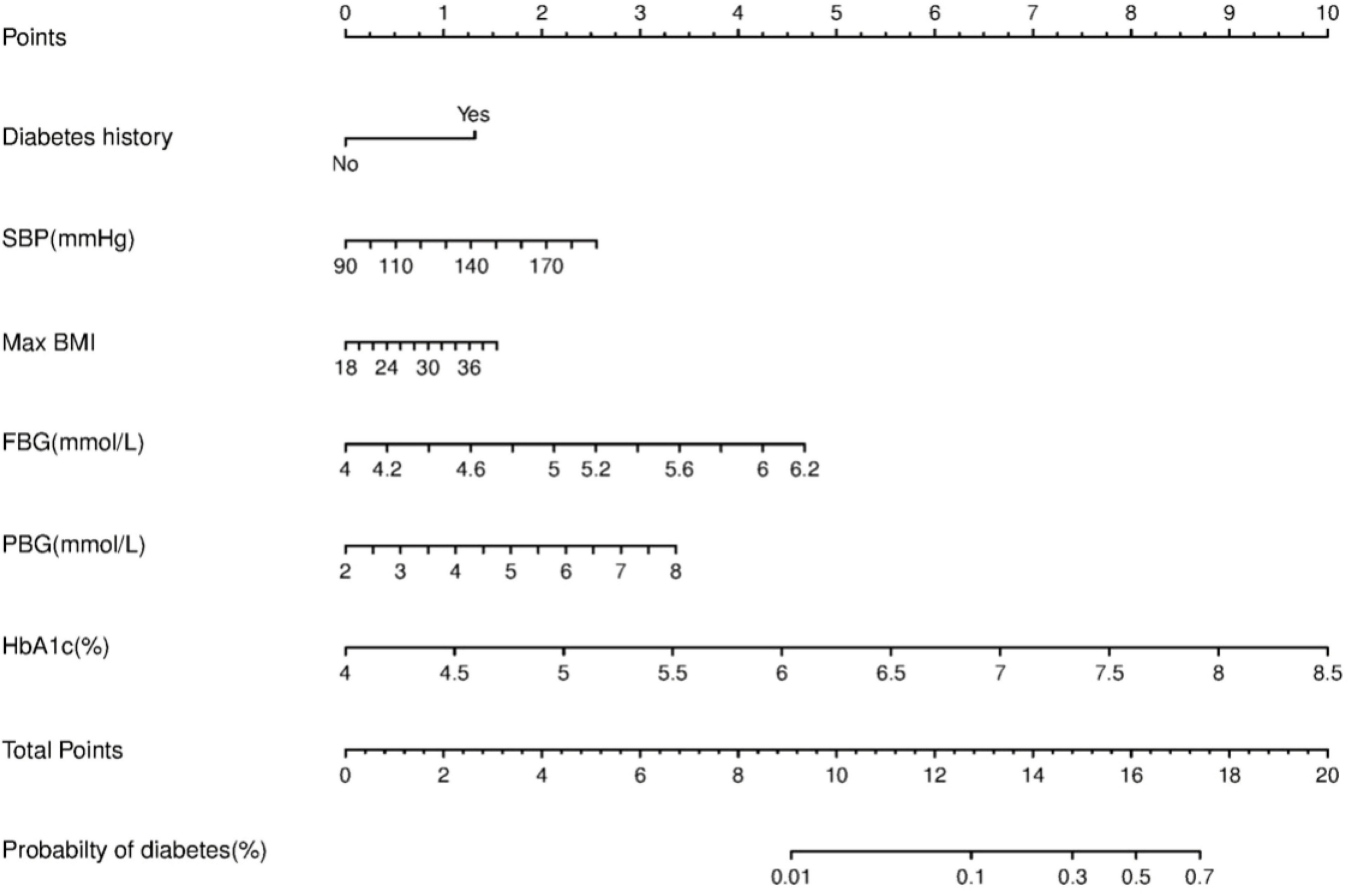
Nomogram predictive model for diabetes in males.

We constructed a nomogram for diabetes according to the variables in **Table 2**, and the probability of diabetes in subjects was evaluated by summing the scores for each variable (**Figure 1**). The risk of diabetes predicted by this nomogram ranged from 0.01 to 0.7. HbA_1c_ was related to the highest risk of diabetes, followed by fasting plasma glucose, SBP, BMImax, and familial history. A sex-specific predictive model is shown in **Figures 2**, **3**.

**Table 2:** Crude and multivariable-adjusted relative risk of related factors for diabetes.

	Unadjusted		Adjusted	
	OR (95%CI)	P-value	OR (95%CI)	P-value
Whole participants				
BMI max		0.001		0.002
normal	1.0		1.0	
Overweight	1.24 (0.72-2.12)	0.442	1.13 (0.57-2.23)	0.729
Obese	2.52 (1.45-4.37)	0.001	3.26 (1.42-7.52)	0.006
Fasting plasm glucose	6.30 (3.47-11.44)	0.000	3.69 (1.93-7.07)	0.000
2h post load glucose	1.50 (1.20-1.87)	0.000	1.37 (1.08-1.75)	0.010
HbA_1C_	3.54 (2.34-5.36)	0.000	2.66 (1.67-4.23)	0.000
SBP	1.01 (1.00-1.02)	0.004	1.03 (1.01-1.05)	0.002
HDL-c	0.40 (0.22-0.74)	0.003	0.48 (0.24-0.97)	0.042
LDL-c	1.34 (1.07-1.68)	0.011	1.20 (0.92-1.55)	0.178
Family history of diabetes	2.34 (1.54-3.54)	0.000	1.94 (1.25-3.01)	0.003
Male				
Fasting plasm glucose	9.89 (3.32-29.5)	0.000	5.26 (1.57-17.64)	0.007
2h post load glucose	1.63 (1.14-2.33)	0.007	1.53 (1.03-2.28)	0.036
HbA_1C_	6.08 (2.81-13.14)	0.000	4.39 (1.96-9.82)	0.000
Family history of diabetes	2.93 (1.45-5.92)	0.003	2.34 (1.09-5.01)	0.029
Female				
BMI max		0.001		0.006
normal	1.0		1.0	
Overweight	1.03 (0.54-1.97)	0.927	1.06 (0.47-2.38)	0.894
Obese	2.78 (1.45-5.45)	0.002	3.97 (1.38-11.44)	0.011
Fasting plasm glucose	5.08 (2.48-10.4)	0.000	3.34 (1.49-7.45)	0.003
HbA_1C_	2.78 (1.67-4.65)	0.000	2.02 (1.13-3.62)	0.018
SBP	1.01 (1.00-1.02)	0.022	1.01 (1.00-1.02)	0.034
HDL-c	0.28 (0.13-0.62)	0.002	0.38 (0.16-0.91)	0.030
Family history of diabetes	2.12 (1.27-3.54)	0.004	1.85 (1.07-3.18)	0.027

Odds ratio with 95% CI are shown for diabetes. In whole population, multivariate analyses adjusted for age, sex, waist to hip ratio, baseline BMI, BMImax, systolic blood pressure, fasting plasm glucose, 2h post load glucose, HbA_1C_, HDL-c, LDL-c, family history of diabetes. In male, multivariate analyses adjusted for age, fasting plasm glucose, 2h post load glucose, HbA_1C_, family history of diabetes. In female, the adjusted model included BMImax, fasting plasm glucose, HbA_1C,_ systolic blood pressure, HDL-c, family history of diabetes. BMImax, Maximum body mass index; HbA_1C_, Glycosylated hemoglobin; SBP, Systolic blood pressure; HDL-c, High‐density lipoprotein cholesterol; LDL-c, Low-density lipoprotein cholesterol.

ROC curves and decision curve analysis (DCA) were used to verify the accuracy of the scoring system. The area under the curve (AUC) was 0.778 (95% CI: 0.733–0.823, *P*<0.001) in all individuals, 0.813 (95% CI: 0.738-0.889, *P*<0.001) in males and 0.759 (95% CI: 0.701-0.817, *P*<0.001) in females. DCA was used to evaluate the discriminative ability of the nomogram. The vertical axis is the net benefit (NB), and the horizontal coordinate axis X is the threshold probability. The solid black line was complete nonintervention (None) with an NB of zero. The gray dotted line indicates complete intervention (All). The results of DCA indicated that the predictive model achieved a greater NB than the None or All models (data not shown).

## Discussion

Our study of Chinese individuals demonstrated that obesity defined by BMImax was positively related to the risk of progression to diabetes, especially for females. BMImax, could be regarded as an independent predictor in the screening and prevention of diabetes in high-risk populations. To our knowledge, this is the first study to explore the association between the historical BMImax and the risk of diabetes in a Chinese population.

The relationship between obesity and diabetes has been reported in several previous studies. Most of the research focused on body weight or body weight change at a certain time point during the research period. Few studies have evaluated the effect of highest lifetime BMI on the progression of diabetes. In 2014, Yoshizawa examined current BMI and BMI history as screening indicators for diabetes. In that study, individuals in the highest tertile of BMImax (≥26.09 kg/m^2^ in men and ≥24.27 kg/m^2^ in women) had a significantly higher risk for diabetes than those in the lowest tertile (18). Recently, a study from Japan investigated the clinical significance of BMImax before the onset of type 2 diabetes for predicting pancreatic beta cell function. They found that BMImax was an independent factor indicating beta cell function and could predict insulin secretion capacity at diagnosis. However, it did not affect the rate of decrease in islet secretion function after a diagnosis of diabetes (13). Several fundamental studies have also explored the close link between obesity and T2DM. In 2014, Mallafre reported that signaling of glucose-dependent insulinotropic peptide (GIP) and its receptor (GIPR) played key roles in the pathogenesis of obesity and its associated insulin resistance. GIP has important metabolic actions other than its physiological glucose-dependent incretin effect. Particularly in human adipose tissue, GIP might act as an insulin sensitizer and an anti-inflammatory factor. Insulin-resistant states related to obesity might be associated with diminished GIP sensitivity, and GIPR expression in human adipose tissue might determine systemic insulin sensitivity (19). Recently, a study provided compelling evidence showing that an inverse correlation between increasing body mass index and decreasing insulin receptor expression in visceral adipose tissue. Visceral adipose tissue-specific downregulation of the insulin receptor was found to be an early event in obesity-related adipose cell dysfunction, which increases systemic insulin resistance in obese humans (20). As insulin resistance and beta cell dysfunction were found to be the main pathogenic mechanisms of T2DM, both of these effects caused by the highest lifetime BMI might influence the progression of diabetes.

Our study suggested that the association between BMImax and diabetes is sex-specific, in accordance with previous research. A study published in PLOS Genetics revealed sex differences in the effect of obesity traits on the risk of type 2 diabetes—a higher BMI led to a higher risk of type 2 diabetes in women than in men. In addition, Logue reported that the mean BMI closest to the date of diagnosis of type 2 diabetes was 31.83 kg/m2 in men and 33.69 kg/m2 in women, so the inverse relationship between BMI and type 2 diabetes was significantly steeper in women than in men (21). A study published in Diabetologia noted the effect of historical weight on glucose metabolism in women. This study was designed to explore the association of the duration of obesity and cumulative exposure with disease. The results indicated that age at onset of obesity was negatively associated with type 2 diabetes in a cohort of women aged 18-23 years at baseline, and the duration of obesity was positively related to type 2 diabetes (22). In contrast, according to the report of Yutaka, the effect of BMI on diabetes in men and women might be related to genetic susceptibility. The authors analyzed the correlations of single-nucleotide polymorphisms (SNPs) of the FTO gene, current BMI, BMImax and type 2 diabetes, and found that SNPs within FTO influence the development of type 2 diabetes by acting on BMImax. However, the result was not obvious in women (23). The researchers attributed the sex-specific effect to the differences in fat distribution and/or physical activity between men and women, which might influence the effects of FTO variants on obesity/adiposity. However, that study was a cross-sectional study with a small sample. The differences in the metabolic effects of obesity between different sexes might be related to estrogen. The ventromedial hypothalamus plays chief roles in regulating energy and glucose homeostasis and is sexually dimorphic. Research has reported the sex-specific role of GLUR5 in regulating the firing rate, intrinsic excitability, and excitatory and inhibitory transmission in ventromedial hypothalamic (VMH) SF1 neurons in female mice, facilitating glycemic control and lipid metabolism. Furthermore, the study also found that intact mGluR5 function in SF1 neurons is required for estrogen to achieve its positive effects on VMH neuronal activity and glucose homeostasis (22).

In addition to BMImax, our results suggested that fasting plasma glucose, 2 h postload glucose and HbA_1c_ could predict the risk of diabetes. However, the correlation among fasting plasma glucose, HbA_1c_ and diabetes was much stronger than that among 2 h postload glucose. In line with our results, two cohort studies proposed the predictive value of fasting glucose and HbA_1c._ Yoriko suggested that an HbA_1c_ of 5.7%-6.4% could be regarded as an indicator of diabetes, with a value similar to that of fasting glucose. The two tests used together could efficiently target people who are most likely to develop diabetes (24). Heianza’s research reported that the combination of an HbA_1c_ of 5.7%-6.4% and a fasting glucose of 5.6-6.9 mmol/l had the best performance in reducing the likelihood of missing future cases of diabetes. An HbA_1c_ of 6.0%-6.4% and a fasting glucose of 6.1-6.9 mmol/l could be used to screen for diabetes among prediabetic individuals (24). As glucose tolerance tests are not generally conducted during routine medical examinations, fasting glucose combined with HbA_1c,_ which could reflect chronic hyperglycemia and better evaluate the potential risk of impaired glucose regulation.

Nomograms provided a statistical predictive model that includes simple scores of different clinical indicators in a visual and quantitative way and have been widely used in the evaluation of the risk and possibility of specific diseases or clinical events. Considering that factors associated with diabetes vary by sex, we made separate predictive models for males and females. The variables included in the nomograms were accessible in clinic and could provide a reference for clinicians to assess the risk of diabetes for different patients, facilitating individualized clinical management. In our study, HbA_1c_, followed by fasting glucose, was the most effective predictor among both men and women. BMImax and postload glucose ranked next in effectiveness. Consistent with a recently published study in a Chinese population, HbA_1c_ could improve the performance of nomograms for predicting the 5-year incidence of type 2 diabetes (25). Although we innovatively incorporated BMImax into the predictive model, the efficacy of the nomogram assessed by the ROC curve was moderate, which might be related to the following factors. The effect of obesity on the prevalence of diabetes might be exerted in several ways, for example, fluctuations in weight, highest weight in adulthood, or even the duration. The predictive model developed in this study explored only the effect of historical highest BMI on the risk of developing diabetes. If we could have included more obesity-related factors, such as those mentioned above, a more comprehensive analysis of the effect of body weight on glucose metabolism might have been possible. In addition, the 92 patients who developed diabetes in this study did not include patients with reduced glucose tolerance and impaired fasting glucose regulation, thus failing to mention the effect of maximum body weight on prediabetes.

Our study focused on the correlation between BMImax and diabetes. Several limitations should be considered in this study. First, the definitive types of diabetes were not clear due to unavailable clinical evidence, including diabetes-associated autoantibodies. We hypothesized that our conclusions could be extended to individuals with type 2 diabetes. Second, lifetime BMImax before the onset of diabetes was self-reported and inevitably had a certain recall bias. Further studies should be conducted to confirm these findings with actual measurements of body weight and height. To preliminarily investigate whether recall bias might affect the findings of our study, we divided the subjects into two groups according to the time of BMImax. One group of subjects reached maximum weight at baseline. Subjects in the second group reached their maximum weight before the onset of the study, and thus BMImax was obtained by self-report. We found a correlation between BMImax and diabetes in both groups.

In conclusion, obesity defined by lifetime BMImax has an obvious association with the risk of diabetes in Chinese individuals over 40 years old, especially women. Given the ongoing increases in the prevalence of diabetes and obesity, a better understanding of the effect of obesity is certainly needed to improve the prevention and management of diabetes and diabetes-related complications.

## Data Availability Statement

The original contributions presented in the study are included in the article/supplementary material. Further inquiries can be directed to the corresponding authors.

## Ethics Statement

The studies involving human participants were reviewed and approved by Clinical Research Ethics Committee of the people’s Liberation Army General Hospital. The patients/participants provided their written informed consent to participate in this study.

## Author Contributions

XJ, AW and ZL designed the study. XJ, AW, LY, YC, YW collected and analysed the data. JB, JD, YM and DZ contributed samples for this study and provided intellectual input. XJ drafted and wrote the manuscript. AW, DZ and ZL performed critical revisions to the manuscript. All authors contributed to the article and approved the submitted version.

## Funding

This work was supported by a grant from the National Key Research and Development Program of China (2018YFC1314100).

## Conflict of Interest

The authors declare that the research was conducted in the absence of any commercial or financial relationships that could be construed as a potential conflict of interest.

## Publisher’s Note

All claims expressed in this article are solely those of the authors and do not necessarily represent those of their affiliated organizations, or those of the publisher, the editors and the reviewers. Any product that may be evaluated in this article, or claim that may be made by its manufacturer, is not guaranteed or endorsed by the publisher.
